# Performance of microvascular anastomosis with a new robotic visualization system: proof of concept

**DOI:** 10.1007/s11701-021-01294-5

**Published:** 2021-08-19

**Authors:** F. Boehm, P. J. Schuler, R. Riepl, L. Schild, T. K. Hoffmann, J. Greve

**Affiliations:** 1 Department of Otorhinolaryngology, Head and Neck Surgery, Ulm University Medical Center, Frauensteige 12, 89075 Ulm, Germany; 2 Surgical Oncology Ulm, i2SOUL Consortium, Ulm, Germany

**Keywords:** Exoscope, Microvascular flap, Microvascular anastomoses, Head and neck surgery, Micro-surgery, Robotics, Ergonomics

## Abstract

Microvascular procedures require visual magnification of the surgical field, e.g. by a microscope. This can be accompanied by an unergonomic posture with musculoskeletal pain or long-term degenerative changes as the eye is bound to the ocular throughout the whole procedure. The presented study describes the advantages and drawbacks of a 3D exoscope camera system. The RoboticScope^®^-system (BHS Technologies^®^, Innsbruck, Austria) features a high-resolution 3D-camera that is placed over the surgical field and a head-mounted-display (HMD) that the camera pictures are transferred to. A motion sensor in the HMD allows for hands-free change of the exoscope position via head movements. For general evaluation of the system functions coronary artery anastomoses of ex-vivo pig hearts were performed. Second, the system was evaluated for anastomosis of a radial-forearm-free-flap in a clinical setting/in vivo. The system positioning was possible entirely hands-free using head movements. Camera control was intuitive; visualization of the operation site was adequate and independent from head or body position. Besides technical instructions of the providing company, there was no special surgical training of the surgeons or involved staff upfront performing the procedures necessary. An ergonomic assessment questionnaire showed a favorable ergonomic position in comparison to surgery with a microscope. The outcome of the operated patient was good. There were no intra- or postoperative complications. The exoscope facilitates a change of head and body position without losing focus of the operation site and an ergonomic working position. Repeated applications have to clarify if the system benefits in clinical routine.

## Introduction

The ultimate goal of oncologic surgery is the complete resection of malignant tumors with maximal surgical safety. In the field of head and neck surgery extended resections are often challenging as important anatomic structures are located in narrow spaces. Therefore, extensive and safe tumor resections are often associated with considerable loss of function especially concerning swallowing, vocalization and enunciation [1]. Often complex reconstructions are necessary to compensate or vindicate the loss of function and treat the extensive tissue defects after primary resection.

In the past decade, numerous advances have been achieved in the reconstructive surgery of the head and neck. Especially microvascular free flaps show good outcomes concerning the preservation of function and restoring of appearance, with free flap survival rates exceeding 95% in most surgical departments [2, 3].

Performing microvascular anastomosis requires visual magnification of the surgical field. Usually, this is achieved using a microscope or magnifying spectacles. Using a microscope is combined with an unergonomic position as the eye is bound to the oculars throughout the whole procedure. In consequence, surgeons may suffer from musculoskeletal pain or stiffness in the neck and consequently reduced ability to concentrate and in the long-term degenerative changes of the cervical spine result. A study by Khansa et al. showed that 90 percent of all surgeons suffer from pain and stiffness in the neck; in 27 percent the pain occurred during or after microscope use [4].

**Fig. 1:**
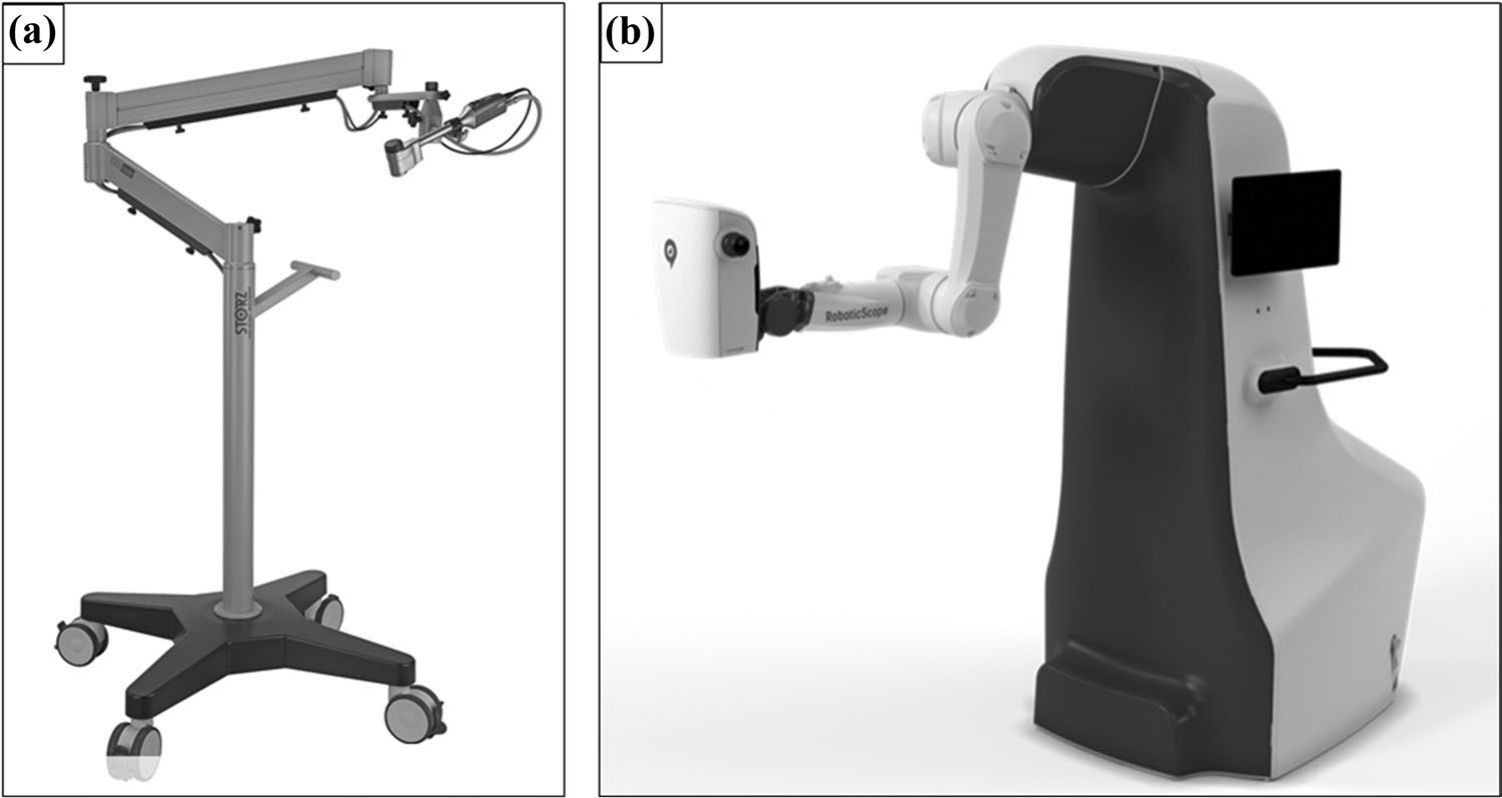
**a** Conventional manual adjustable exoscope, the VITOM^®^ 3D mounted on the VERSACRANE™ light manual holding arm (Karl Storz^®^, Tuttlingen, Germany). Reproduced with kind permission of KARL STORZ SE & Co. KG. **b** Robotic exoscope system, the RoboticScope^®^ (BHS Technologies^®^, Innsbruck, Austria)

Exoscopes can provide a solution to this matter. They are defined as high-resolution cameras which can be placed over the surgical field and transfer the image to a large display. This way the surgeon is free to change his body position without disrupting the view of the operation site [5]. The first time the use of an exoscope in microvascular free flap surgery has been described in 2017 by Piatkowski [6]. Figure 1a shows a conventional manual adjustable exoscope, exemplarily the Vitom 3D^®^ (Karl Storz^®^, Tuttlingen, Germany). This exoscope is also available with a motorized holding arm (ARTip cruise) that can be controlled with a 3D computer mouse (IMAGE1 PILOT) and enables an easy adjustment of the exoscope position.

In the following, we present the clinical application of a robotic exoscope, the RoboticScope^®^ (BHS Technologies^®^, Innsbruck, Austria) (Fig. 1b) in the surgical reconstructive therapy of head and neck cancer as a proof-of-concept assessment. Two other working groups previously described surgeries with the aid of the robotic scope system, one for the performance of a lymphovenous anastomosis [7] and another for a tympanoplasty in a patient with a subtotal tympanic membrane perforation [8]. To the best of our knowledge, we are the first to describe the use of the system in oncologic reconstructive surgery of the head and neck.

## Material and methods

### Materials

**Fig. 2:**
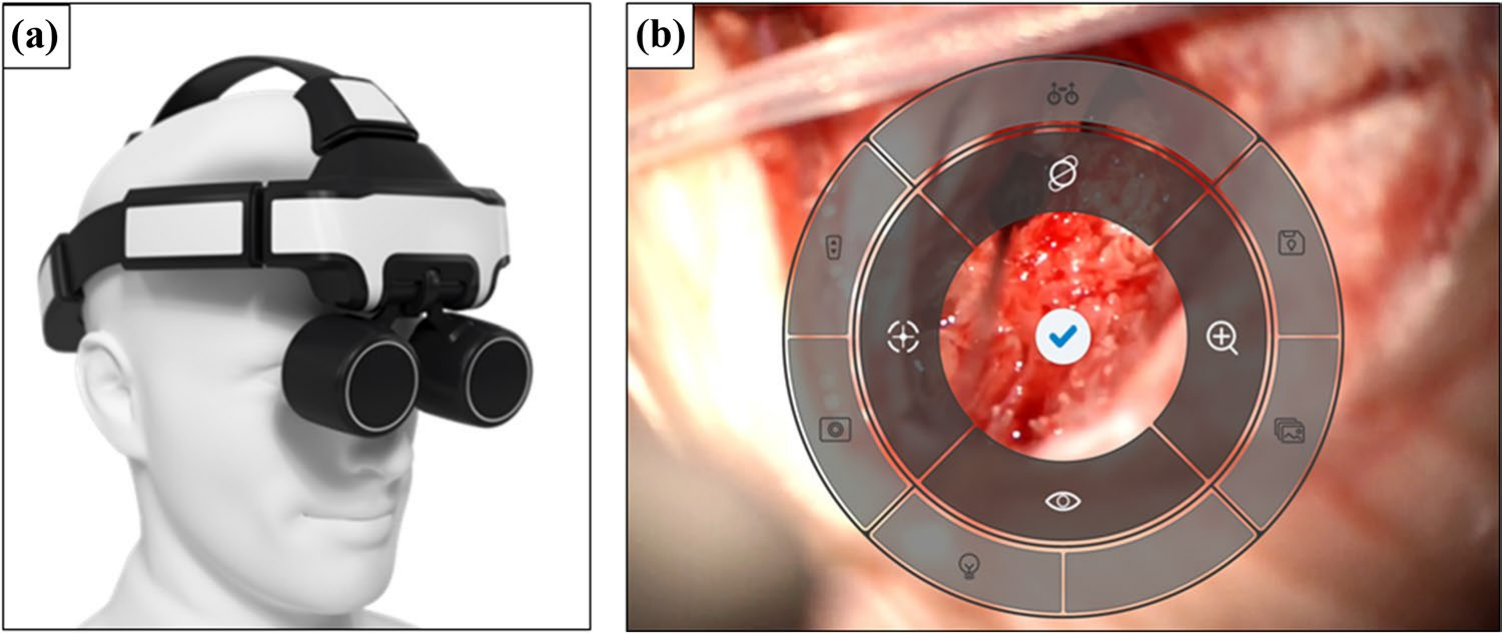
RoboticScope system setup: **a** head-mounted-display (HMD), **b** control menu visible in the HMD after unlocking via foot pedal

The RoboticScope consists of a high-resolution three-dimensional camera with an extended full HD resolution of 4112 × 1542 pixels. The camera is installed on a robotic arm with 6 axes that operates with an accuracy of 0.03 mm and a maximum speed of 250 mm/s. The acquired pictures are transferred to a head-mounted-display (HMD) (Fig. 2a) and an external display. The head-mounted display weighs approximately 0.5 kg. It consists of two digital microdisplays. The pupillary distance can be changed manually according to the surgeon's need. The HMD is connected to the exoscope through a cable connection. A foot pedal unlocks a control menu with various functions. The menu can be operated by the surgeon performing head movements, which are detected by a motion sensor incorporated in the HMD (Fig. 2b). The design of the HMD resembles digital magnifying glasses. These glasses contain a camera and a display included all in one device. The HMD shows pictures of a camera mounted on a robotic exoscope arm that is positioned above the operation site. This allows the surgeon, in contrast to conventional magnifying glasses, to change body and head position freely without disrupting the visualization of the operation site. A visual line of view to the surgical field is not necessary when using the HMD. The camera system of the RoboticScope enables a magnification factor from 2.7 to 30.1 × and a field of view range from 5.8 × 4.3 mm to 64.5 × 48.4 mm with full optical zoom. This allows for an overview as well as a detailed portrayal of the operation site. The surgeon can change the view in the horizontal plane, change the view angle and thus focus and zoom of the camera in the control menu of the HMD solely with head movements. Furthermore, for a better visual impression, the light intensity can be changed and the settings of the HMD-eyepieces can be adapted to different areas of application using the HMD control menu. Additionally, the 3D view can also be changed to a 2D view. This is especially useful in narrow surgical fields with deep cavities, as the visual impression resembles that of an endoscope and facilitates orientation. Apart from operation site visualization, the RoboticScope includes several other features that can also be accessed through head movements. Exemplarily, the eyepieces of the HMD can be lifted fully motorized to get a view of the surroundings and the current robot arm position can be saved and later on restored using the function “Store position”. Furthermore, the system allows to record and save videos or pictures on an external storage device like USB hard drives or memory sticks. The operation time can be recorded and previously recorded files can be viewed.

### Methods

**Fig. 3:**
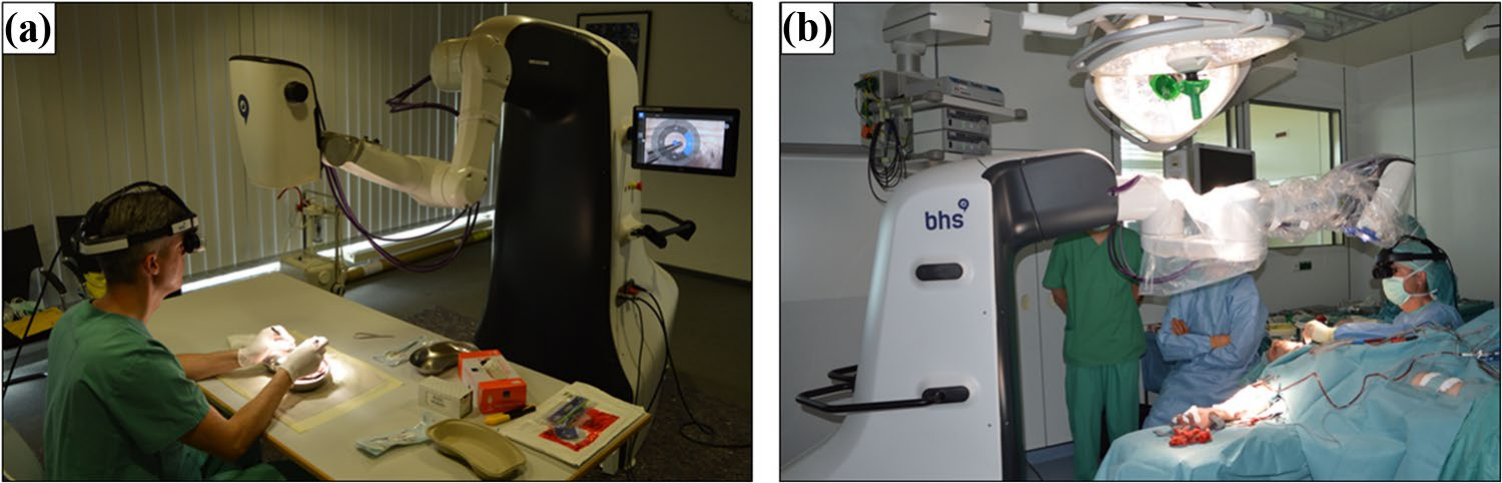
**a** Experimental preclinical setup of the RoboticScope System for testing coronary anastomoses in ex-vivo pig hearts. **b** Operating theatre setup of the RoboticScope System for the anastomosis of a radial free flap in a patient with lateral tongue resection

First, two head and neck oncologic surgeons, experienced in microsurgical techniques, evaluated the feasibility and safety of the presented system for use in microvasculature anastomosis in an experimental ex vivo setup using two pig heart specimens. Both surgeons underwent technical instruction from the providing company beforehand of the procedure. Figure 3a shows the experimental setup. The surgeons first cut the right and left coronary artery and performed an anastomosis using single-button sutures with the RoboticScope setup. Conventional micro needle holders, micro tweezers and a monofilament, non-absorbable suture of the size 8–0 were used. The surgeons examined the different advertised functions of the system, the imaging quality and the safety for application in vivo.

Second, the same surgeons applied the RoboticScope for the surgery of a patient with squamous cell carcinoma of the lateral tongue. Besides the technical instructions already mentioned, there was no special surgical training of the surgeons or involved staff upfront performing the procedure. Figure 3b shows the operating room setup. Extensive tumor resection with expansion across the midline of the tongue was necessary to remove the whole extension of the tumor with safety margins. After transoral tumor resection, the tissue defect was sealed using a radial forearm free flap with the size of 4 × 6 cm and the RoboticScope System was used for visualization and performance of the anastomosis. The anastomosis of the superior thyroid artery and the radial artery was done in end-to-end technique using single-button sutures with a monofilament, non-absorbable suture of the size 8–0. Furthermore, anastomosis of the venous confluency of the accompanying veins to the internal jugular vein was performed in an end-to-side technique using a continuous suture and a monofilament, non-absorbable suture of the size 8–0. One surgeon conducted the arterial and one the venous anastomosis. We measured the setup time of the system as well as the duration time of the arterial and venous anastomosis. For comparison, we quantified the average times needed in comparable surgeries conducted with a conventional microscope. Additionally, we evaluated the ergonomic advantages of the system compared to a microscope relating to the performed surgery through an ergonomic assessment questionnaire, the rapid upper limb assessment (RULA). The patient outcome was evaluated daily during hospitalization and continuously every 3 months after hospital discharge.

## Results

**Fig. 4:**
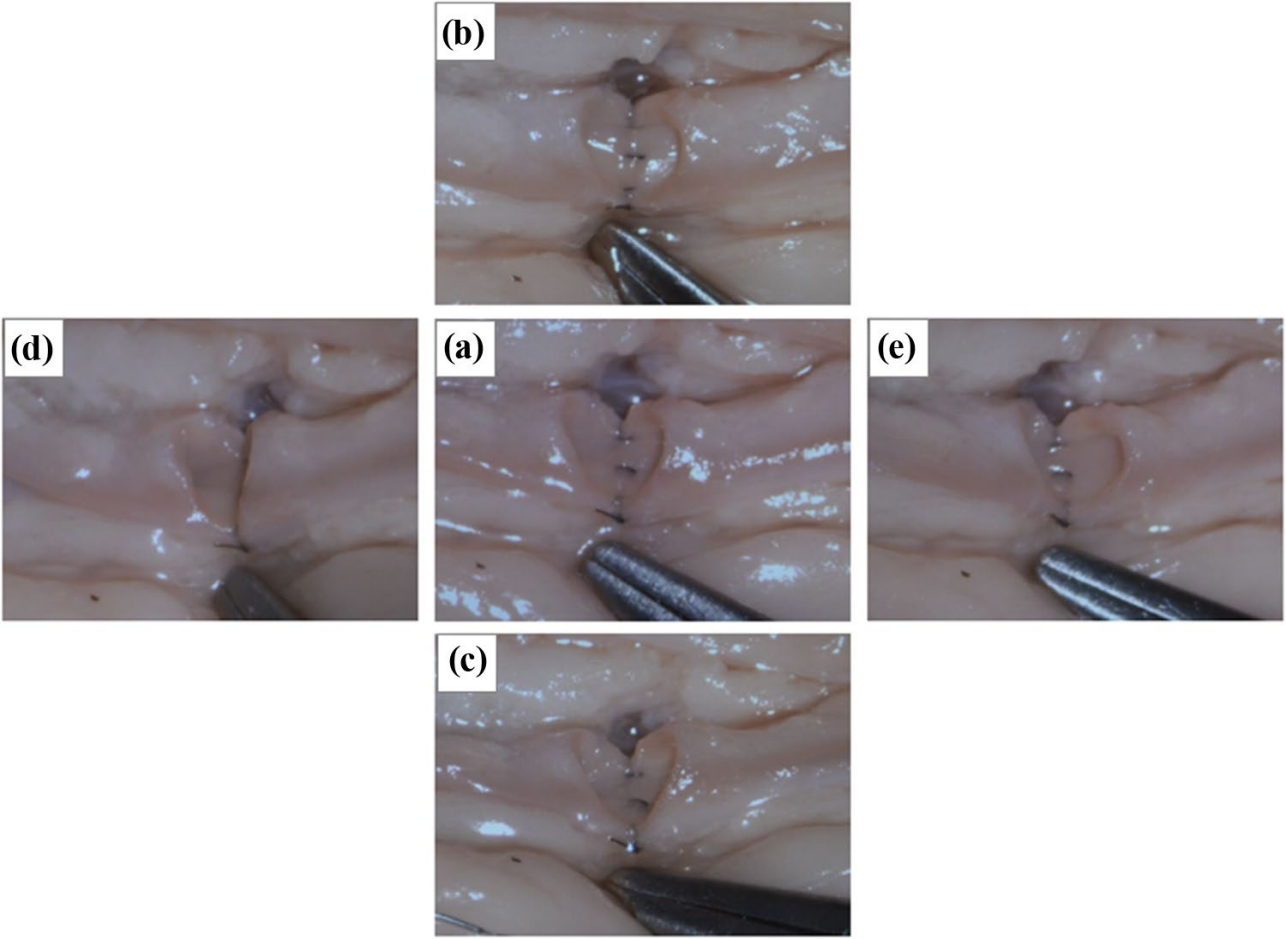
Demonstration of the system function “OrbitView”. **a** Primary output image of the HMD. **b** + **c** View angle from cranial and caudal. **d** + **e** View angle from the right and left side

**Fig. 5:**
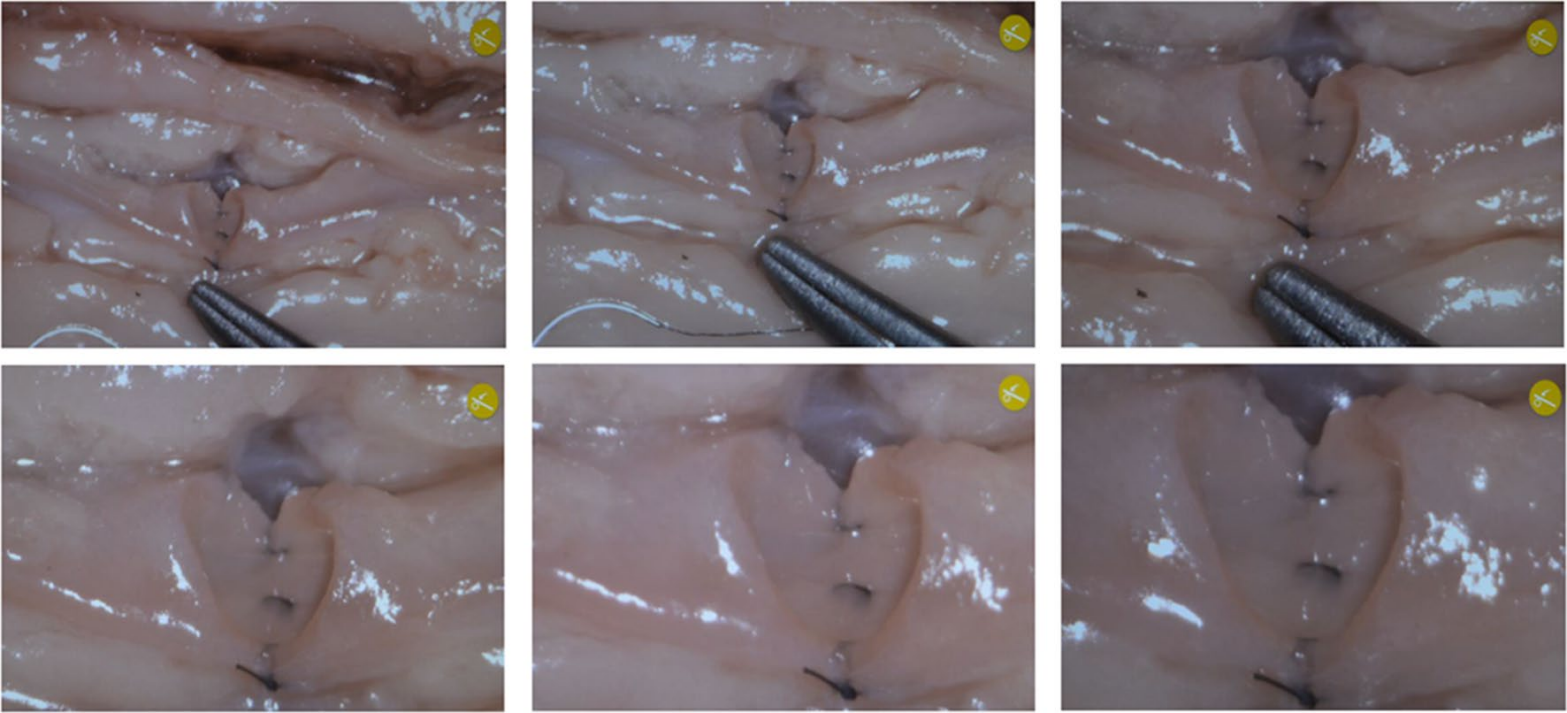
First six degrees of magnification exemplarily depicting the anastomosis of the coronary artery of a pig heart

First, the surgical setup was tested ex vivo by performing coronary artery anastomoses in a pig heart. The positioning of the RoboticScope system was convenient. The robot arm could be set to a basic position by pressing a single button. Manual basic positioning of the system was practical and possible without much effort. The HMD was adaptable to the surgeons’ head circumference and pupillary distance. The access and manipulation of the HMD control menu were intuitive. Through the function “OrbitView” the alteration of view angles could easily be controlled via head movements, and the body position could be maintained during the whole procedure. The function enabled different view angles and directions without losing focus of a previously focused area. Exemplarily, this made it possible to view alternating the lumen of both coronary arteries for the anastomosis. Figure 4 demonstrates the application of the “OrbitView”. The possible range of magnification enabled a detailed depiction of the microvascular vessels, which had in the case of the pig heart a diameter of 2 mm and in the case of a radial artery of 3 mm as well as a good overview of the operation site. The image resolution was good, and high degrees of magnification did not result in a notable loss of resolution due to the full optical zoom. Figure 5 depicts different degrees of magnification, showing exemplarily the coronary artery anastomosis of a pig heart. For these diameters only the low up to the middle degrees of magnification were recommendable as larger magnification impaired orientation.

During the procedure, we noticed an unnatural paleness of the visualized tissue, a change of color settings for a more vivid and natural imaging was not possible. The light intensity could be changed in fine nuances. The picture of the operation site occasionally lacked brightness despite full illumination of the operation site. Real-time picture transfer to the system’s external display was possible without any problem. Transfer to another external display that is not part of the RoboticScope System was not possible at the time of testing.

After the successful approach ex vivo, the system was transferred to clinical/in vivo testing. The previously described surgery in a patient with squamous cell carcinoma of the lateral tongue was performed. The preparation of the surgical setup was simple and took 9.47 min. This included the sterile covering of the system, the booting of the system, connecting the HMD to the system and adjusting the HMD to the surgeon’s head. The sterile cover could be pulled over easily as the robot arm can be extended upon the push of a single button. Due to the design of the robotic arm, the system could be placed easily and without interference with any other surgical systems (Fig. 3b).

**Fig. 6:**
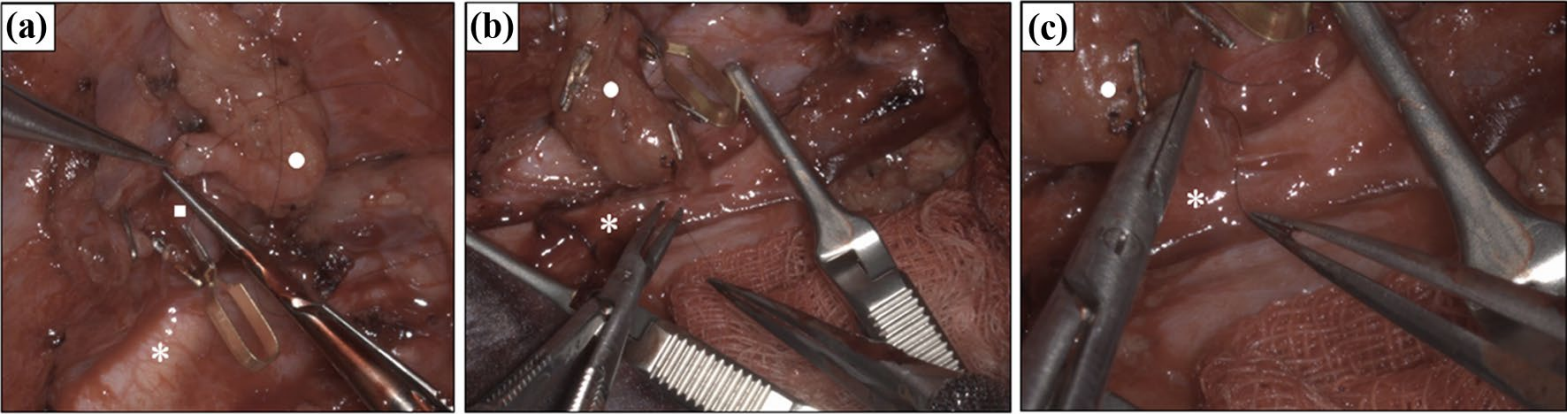
**a** Suturing of the arterial anastomosis of a radialis free flap with the aid of the RoboticScope system. *External carotid artery, □ superior thyroid artery, ○ pedicle of the free flap (**b** + **c**) Performance of the venous anastomosis of a radialis free flap with the aid of the RoboticScope system. **b** Low degree of magnification for a good overview; **c** moderate degree of magnification for the preparation of the venous vessel., *internal jugular vein, ○ venous confluency of the flap veins

The preparation of the arteries and the anastomosis suturing technique could be executed without difficulty. The arterial anastomosis (Fig. 6a) required 22.83 min, and the venous anastomosis (Fig. 6b, c) was performed in 32.03 min. Both time spans include the vascular suture as well as the preceding preparation of the blood vessels. The average procedure times in our clinic for radialis flap anastomosis with a conventional microscope was 70 ± 20 min combined for arterial and venous anastomosis.

No musculoskeletal pain or stiffness in the neck was experienced during or after the procedure. The HMD was not too heavy and was comfortable to wear at all times. The view of the surgical site was comparable to that through a microscope. The simple change of the zoom and the sharpness by moving the head was much more pleasant compared to the microscope since the operational flow was not interrupted by putting the surgical instruments aside. The motorized folding up and down of the HMD, also by moving the head, made it possible to switch quickly between the general overview and magnification. When using a microscope, this has to be pushed aside and repositioned and the focus readjusted when it is used again.

The ergonomic assessment of the surgeons’ posture during the use of the RoboticScope with the Rapid Upper Limb Assessment (RULA) was determined with 2 points accounting for a good ergonomic position of arm, wrist, neck, trunk and legs with no need for improvements.

The ergonomic assessment of the surgeons’ posture during the use of a conventional microscope with the RULA was determined with 3–4 points accounting for a critical working posture with the recommendation on further investigation and the implication of ergonomic improvements.

There were no complications during or after the surgery. In early post-surgery treatment, the free radial flap was vital, there was no dehiscence and the patient was able to move the remnants of the tongue. 2 weeks after the surgery the swallowing of soft food was feasible. The patient could be transferred from the intensive care unit (ICU) after 9 days to the general ward. The main reason for the 9-day-long ICU treatment was postoperative delirium as a consequence of chronic alcohol abuse, not the surgical procedure itself. The overall duration of the hospital stay was in total 19 days. Seven months after surgery the free radial flap is still vital and in place. The patient is currently disease-free.

## Discussion

Ex vivo testing revealed an intuitive and precise handling of the system. The functions described by the providing company could be easily accessed.

The significant difference between microscopic surgical manipulation and manipulation with RoboticScope is the relationship between the visual line and surgical site: in microscopic manipulation, the visual line is directed towards the surgical site [9–11]. When using the RoboticScope, the visual line does not necessarily point towards the surgical site, which is, at first, an unusual situation for the surgeon. In endoscopic sinus surgery, an endoscope camera is usually used and the camera image is transmitted to an external monitor, so that head and neck surgeons are used to the fact that their visual axis does not always point to the operating field [12, 13].

Still, in our opinion, there is no special training needed beforehand the use of the system. There was a steep learning curve concerning the control of the system. Especially surgeons already experienced with the handling and the suturing of an anastomosis with a microscope will be able to easily switch to the RoboticScope system.

In clinical in vivo testing the presented exoscope system setup took considerably longer than the average setup time for a microscope, which is approximately 3–4 min in our department. It has to be taken into account that the surgical staff used the robotic scope system for the first time and that there was no training prior to the procedure. Still, while the setup time of the exoscope will most likely decrease with increasing routine, the time will likely remain longer than the time needed for the setup of a conventional microscope, as the microscope only needs a sterile cover and can then be positioned manually. In comparison, the exoscope needs additional time for booting, manual basic positioning and subsequently digital fine adjustment of the position. Additionally, the HMD must be connected to the system, must be put on by the surgeon and then adjusted to head circumference and pupillary distance.

The RoboticScope allowed for good visualization. The degrees of magnification and the sharpness of view were comparable to a standard microscope. The different view angles and directions generated by the OrbitView allowed for good and individual imaging of the operation site. The OrbitView was permanently in use during the surgery. The possibility to change the viewing angle of the operation site enabled the surgeon to look at the blood vessels that need to be anastomosed from different perspectives and consequently facilitated suturing with optimal view of tissue, needle and thread. A drawback of the images displayed on the HMD and the system’s external monitor was the unnatural paleness of the visualized tissue and the occasional lack of brightness in visualization. These problems are a result of the applied imaging postprocessing algorithms. The absolute brightness of the installed light source, however, is adequate. For outside observers with free view to the operating site, like assisting surgeons, surgical nurses, or medical students the operation site is brightly illuminated. The lack of brightness and the sallow image on the HMD may complicate the suturing of the anastomosis when an undyed or white thread is used for suturing. This was not a problem in our case as already dark-colored suture was used. Despite the lack of brightness different anatomical structures, especially muscles, vessels and nerves could be differentiated safely in the animal experiment as well as in the in vivo testing. As confirmation that the problem is a matter of image processing, the manufacturing company developed a software update that enables more vivid, natural and brighter imaging. In our study, we almost exclusively applied the 3D sight, which allows for a stereoscopic view and consequently for optimal orientation. In the instances that the 2D view was used, we could not detect any relevant advantages of the 2D view in comparison to the 3D view, neither in our ex-vivo experiment nor in the in-vivo application. Admittedly, this is not surprising, because the 2D viewing mode was developed for surgeries in operation sites with deep cavities and the need for high magnification levels. In this setting, the combination of depth and large magnification can potentially complicate intraoperative orientation.

The 2D view mode allows, like described in the chapter material and methods, a view very similar to endoscopic devices which shall facilitate orientation in these distinct surgical settings. As our operation site was easily accessible in our surgical setup and as there were no deep cavities, we could not detect any additional benefit of the 2D view mode in comparison to the 3D mode. The time needed for arterial and venous anastomosis in the experimental setup was comparable to the time required in surgery with a conventional microscope. The flap ischemia time was 69 min for the use of the RoboticScope. For comparison, radial free flap ischemia times in the reconstruction of head and neck tissue defects vary from 56 to 108 min in literature when a conventional microscope is used [14–16]. In our clinic the average flap ischemia time in similar surgical procedures with the use of a conventional microscope was 85 min.

A notable advantage of the RoboticScope was that the handling of the exoscope was possible solely with head movements. No further manual adjustments were needed. This enables a continuous and uninterrupted course of the surgery. In comparison, conventional microscopes often need manual adjustments which make it necessary to occasionally interrupt the surgery and lay aside the surgical instruments. In case of big position changes the surgical nurse might even need to switch places or reposition the instrument table. Because of the OrbitView function integrated into the RoboticScope, this was not necessary for our surgical setup. If free-handed control of the exoscope and the allowance for bimanual uninterrupted surgery can contribute to shorter operation times, has to be evaluated in further clinical trials with larger patient cohorts.

One of the main advantages of the RoboticScope in comparison to a conventional microscope was that the system enabled a neutral and upright posture of the spine during the whole surgery rather than a bent-over position. The surgeon was not bound to a stationary eyepiece and as a consequence was not forced to hold a certain position for a large amount of time. The ergonomic assessment questionnaire showed a good ergonomic result for the Robotic Scope whereas the use of a microscope showed unfavorable ergonomics with a risk of developing musculoskeletal symptoms. This is consistent with results from current literature.

Several studies point to an increased risk of work-related musculoskeletal symptoms, especially in microsurgeons [17–19]. Babar-Craig et al., for example, reported a prevalence of 72% for back and neck pain in head and neck surgeons in the UK [20]. A study of 865 plastic surgeons in the United States, Canada and Norway found that especially using loupes and microscopes was associated with musculoskeletal discomfort. The study identified three main ergonomic reasons for musculoskeletal pain in their study: Hyperflexion of the cervical spine, sustained shoulder elevation and pelvic girdle asymmetry [4]. In a study of microsurgery trainees, it was observed that during the use of a microscope the neck was flexed greater than 10 degrees 88% of the time [20]. This causes strain on the cervical muscles as the head weight increases by 10 pounds for every inch that the head is positioned forward [17]. Another risk factor for the development of musculoskeletal pain is a prolonged static posture during surgery. A study by Yu et al. showed that surgeons remain primarily static (0.3 ± 0.4 movements per minute) while using a microscope compared to 5.5 ± 6.1 movements per minute in times without microscope use [17, 21]. An exoscope system can overcome these ergonomic problems as it allows for a frequent change of position without disrupting the view of the operation site as well as an upright neutral spine posture during the whole surgery. This could be seen in our study as well. Thus, an exoscope system like the RoboticScope can be largely beneficial for the surgeon as it may increase the ability to concentrate as well as avoid long-term degenerative changes in the cervical spine.

However, there are some limitations to the system. The real-time camera picture transfer was only possible to the eyepiece and the system’s own external display. This external display size is similar to an average laptop screen and is, therefore, comparatively small. For visualization of details, enabling larger viewing distance and teaching of residents or students, the possibility of picture transfer to a larger external display with HDMI- or Display-Port-cable would be preferable. This was not yet possible in the former system setup. According to the manufacturer, this function is available in the latest system setup version.

Another drawback of the system in clinical use is that there is only one HMD for the main surgeon available. As the size of the system setup does not allow the placement of an additional conventional microscope, the assisting surgeon must rely on the small external display for visualization. In the future, a second HMD for the assisting surgeon, in, e.g. a master–slave setup, would, therefore, be desirable. Currently, the previously described function with the usage of a second HMD is in preparation and implementation is expected in the near future.

Furthermore, a combination with surgical navigation systems or the possibility to show surgical pathways that were planned before the surgery on the HMD could add additional advantages to the system. In comparison, the most commonly used surgical robots like the DaVinci System were developed primarily for abdominal/pelvic surgery, like urology, visceral surgery and gynaecology. These systems aim to enable a better overview of the abdominal cavity and to improve the handling of the surgical instruments. In ENT surgery these systems exhibit distinct additional benefits only in a small number of patients and in countries with limited experience in transoral laser microsurgery. This is due to the considerable high purchase prices and running costs of robotic systems as well as the lack of surgical instruments small enough to fit the narrow anatomic regions in the head and neck surgery. In contrast, from a financial point of view the RoboticScope requires solely a sterile cover, special surgical instruments are not necessary, which is why the running costs are very low. Additionally, the RoboticScope allows for the use of conventional microsurgical instruments.

There are some limitations to the presented study. As the study only includes one patient case the significance of the retrieved data concerning time measurements and patient outcome is limited. Furthermore, the system was only evaluated by two surgeons and, therefore, the optical impressions, the feasibility of the system handling and ergonomic questionnaire remain subjective assessment. As the system is new and has not been described in the use of oncologic reconstructive surgery of the head and neck before, it was the aim of this study to evaluate the system concerning feasibility, safety concerns and possible advantages as a proof-of-concept assessment. Overall, the system appears promising and displayed several potential advantages in microvascular surgery. If the system indeed leads to a shorter operation time, allows for better visualization, less musculoskeletal symptoms in surgeons or better surgical outcomes will have to be evaluated in larger patient cohorts.

## Conclusion

The preclinical and the clinical application of the RoboticScope exoscope system for microvascular anastomoses showed good results concerning feasibility and visualization. It allowed a hands-free visualization of the operation site during the whole procedure. Operation times were comparable to the duration of surgery using a conventional microscope. The outcome of the treated patient was good. There were no intra- or postoperative complications. The HMD proved especially beneficial for the surgeons as it allowed an ergonomic, upright posture of the spine during the surgery. In comparison to the use of a conventional microscope, which showed unfavorable ergonomic results in the rapid upper limb assessment, the RoboticScope provides an ergonomic working position and can thus reduce the risk for cervical musculoskeletal pain or long-term degenerative changes in the cervical spine.

Further improvements in hardware and software, for example the opportunity to use a second HMD for the assisting surgeon, real-time picture transfer to external displays and the possibility to adjust the colour of the picture settings are desirable for use in clinical routine. Due to the modular construction concept of the system these requirements can most likely be met in the near future.

Our proof-of-concept assessment showed that the RoboticScope is feasible for use in reconstructive microvascular surgery of the head and neck. The testing surgeons experienced several benefits of the system in comparison to a conventional microscope. Advantages concerning operating time, patient outcome and favorable ergonomics will have to be verified in repeated applications, in larger patient cohorts and with a larger number of surgeons, preferably of different levels of experience in microvascular surgery.
